# Development and Characterization of a Benchtop Corneal Puncture Injury Model

**DOI:** 10.1038/s41598-020-61079-y

**Published:** 2020-03-06

**Authors:** Eric J. Snider, Lauren E. Cornell, Jorge M. Acevedo, Brandon Gross, Peter R. Edsall, Brian J. Lund, David O. Zamora

**Affiliations:** Sensory Trauma Research Department, United States Army Institute of Surgical Research, Fort Sam Houston, TX 78234 USA

**Keywords:** Biological models, Biomaterials, Visual system

## Abstract

During recent military operations, eye-related injuries have risen in frequency due to increased use of explosive weaponry which often result in corneal puncture injuries. These have one of the poorest visual outcomes for wounded soldiers, often resulting in blindness due to the large variations in injury shape, size, and severity. As a result, improved therapeutics are needed which can stabilize the injury site and promote wound healing. Unfortunately, current corneal puncture injury models are not capable of producing irregularly shaped, large, high-speed injuries as seen on the battlefield, making relevant therapeutic development challenging. Here, we present a benchtop corneal puncture injury model for use with enucleated eyes that utilizes a high-speed solenoid device suitable for creating military-relevant injuries. We first established system baselines and ocular performance metrics, standardizing the different aspects of the benchtop model to ensure consistent results and properly account for tissue variability. The benchtop model was evaluated with corneal puncture injury objects up to 4.2 mm in diameter which generated intraocular pressure levels exceeding 1500 mmHg. Overall, the created benchtop model provides an initial platform for better characterizing corneal puncture injuries as seen in a military relevant clinical setting and a realistic approach for assessing potential therapeutics.

## Introduction

Eye-related injuries have risen in recent combat operations due to increased use of explosive weaponry. From World War I to Operations Iraqi and Enduring Freedom, eye-related injuries increased from 2% to 13% of combat injuries, respectively^1^. The most prevalent ocular injuries are corneal abrasions, lacerations, and full thickness punctures, resulting in vision impairment without timely intervention^2^. While there are treatments readily available for superficial corneal damage, more severe injuries such as full-thickness corneal punctures (CP) are challenging to treat. CP wounds predominantly enter through the cornea and may damage the iris, lens, and even retina, depending on the speed, material, and trajectory of how the eye was punctured^3^. These injuries are not mitigated until injured warfighters are evacuated back to an ophthalmic specialist for surgical repair. For CP injuries where intraocular foreign bodies are present, the average time between point of injury and surgical repair was 21 days in recent combat operations^4^. An open, untreated CP injury is highly susceptible to infection, metallic poisoning, and reduced intraocular pressure (IOP). Further, low IOP results in poor nutrient delivery throughout the eye, impacting viability and function of ocular tissues^3,5,6^. As a result, CP injuries result in poor visual outcomes even after medical intervention^7^.

To date, when CP injuries occur on the battlefield the chain of treatment first involves placing a rigid eye shield on the injury site until evaluation by an ophthalmic specialist. If no intraocular foreign body (IOFB) is present, sutures are the gold standard for closing these open globe injuries. However, 53% of CP injured eyes retain IOFB upon injury and require evacuation to an ophthalmic specialist for surgical intervention^8^. In these cases, a rigid eye shield may or may not be used depending upon the location of the IOFB, in an effort to minimize disturbance to the damaged area.

Due to the unique nature of these injuries in conjunction with poor visual outcomes, an effort is being made to develop improved therapeutics for CP injuries. However, evaluation of therapeutics for open globe injuries is inadequate due to a lack of military relevant CP models. Previously developed models have been focused on blunt closed-globe corneal injuries or utilized a scalpel to create simple, linear open-globe lacerations^9–14^. Military-relevant injuries are on average 3.5–5 mm in diameter, making them much larger than a scalpel injury^3,4,7^. Here, we demonstrate a benchtop, high-speed open-globe injury model capable of creating military-relevant injuries.

## Results

### CP injury model – System characterization

**Figure 1:**
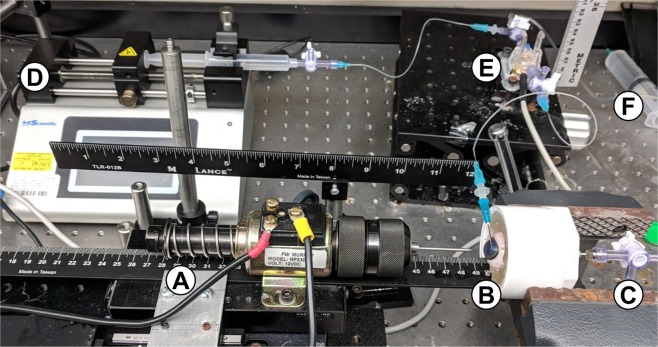
Benchtop Corneal Puncture Injury Model. (**A**) High-speed solenoid puncture device fitted with drill chuck, (**B**) eye holder filled with 10% gelatin (w/v), (**C**) posterior 16-gauge needle for inserting catheter pressure transducer in vitreous chamber, (**D**) syringe pump for volume manipulation, (**E**) in-line pressure transducer connected to eye by 23-gauge needle cannulated into anterior chamber, and (**F**) fluidic connection to hydrostatic reservoir for pressure manipulation.

The benchtop CP injury platform makes use of a high-speed solenoid device to create a military relevant open-globe injury (Fig. 1A). Enucleated eyes are held in 10% w/w gelatin, cannulated with a needle in the anterior chamber that is attached to an in-line pressure transducer, syringe pump, and hydrostatic reservoir (Fig. 1B,D–F). A second needle is inserted into the vitreous cavity and a catheter pressure transducer is drawn into the eye for improved intraocular pressure (IOP) readings (Fig. 1C).

**Figure 2:**
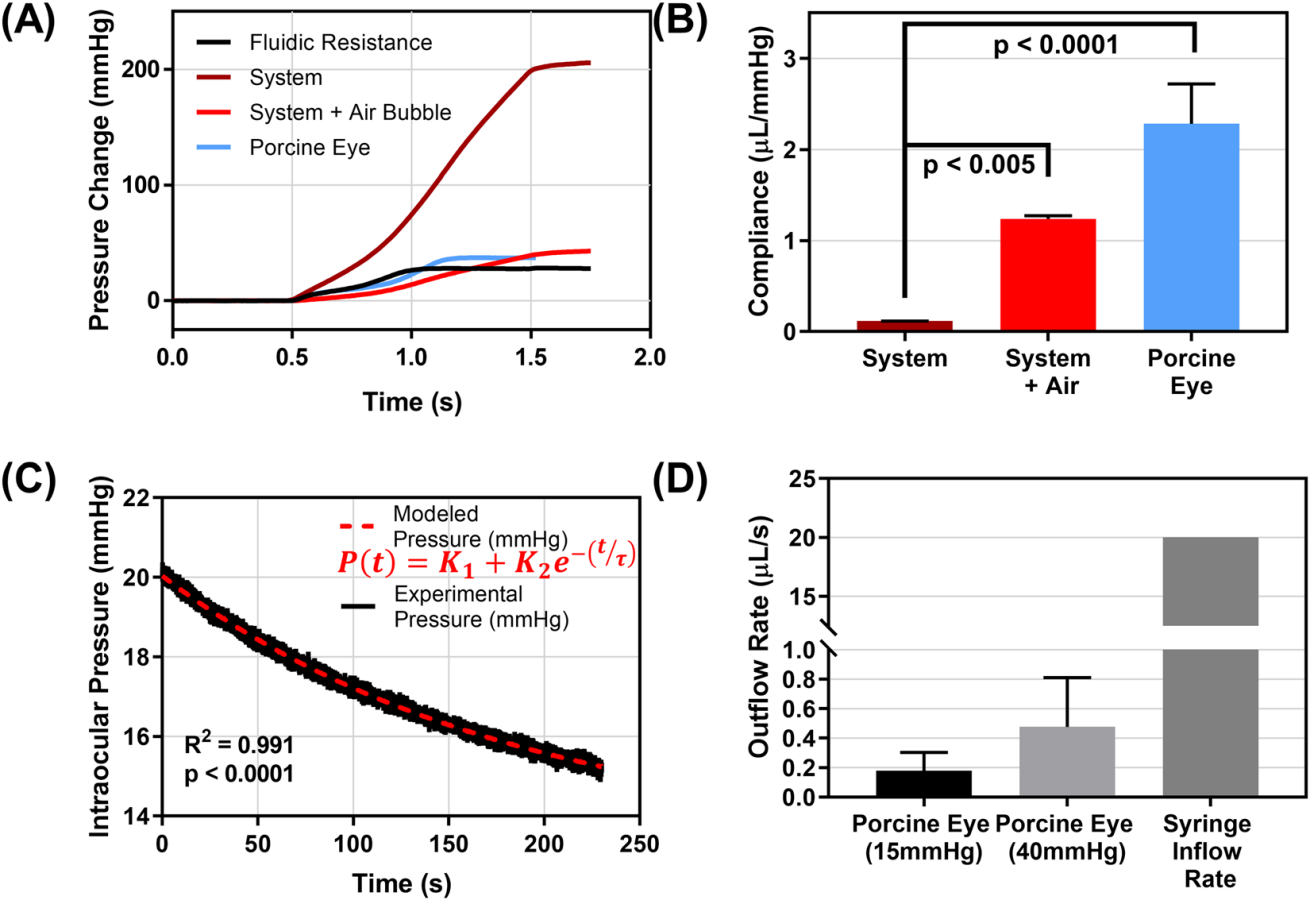
Characterization of compliance and outflow rate in CP injury model. (**A**) Pressure vs. time plot for in-line pressure transducer following a 20 µL injection (20 µl/s flow rate) at 0.5 seconds for the open system (fluidic resistance), normal system, system with an air bubble present, and with a porcine eye attached to the system. (**B**) Compliance values from 20 µL injection for the normal system (n = 3 technical replicates), system containing a single air bubble (n = 3 technical replicates), and a porcine eye attached (n = 3 biological replicates). Significance was determined by one-way ANOVA, post hoc Dunnett’s multiple comparison test. (**C**) Pressure vs. time plot during outflow rate testing as pressure decays from 20 to 15 mmHg. Experimental data and results from an exponential, 3 parameter model. (**D**) Resulting outflow rate for porcine tissue at 15 mmHg and 40 mmHg (n = 15 biological replicates) compared to the 20 µl/s syringe inflow rate. Note that there is a break in the y-axis from 1 µl/s to 10 µL/s to show outflow and inflow rates on the same plot. Error bars denote standard deviation.

In order to standardize the testing platform, pressure readouts and eye properties must remain consistent. We first characterized the compliance of the system to establish baseline parameters for how pressure increases for a given 20 µL infusion (Fig. 2A). Compliance, defined as a change in volume for a given change in pressure, was approximately 0.11 µL/mmHg for the system under normal circumstances (Fig. 2B). To simulate irregular circumstances in the system, we introduced a single air bubble into the system prior to measuring compliance. Given the ease of compressibility of air compared to liquid, a 10-fold increase in compliance was noted after air-bubble introduction. This statistically significant difference can be used to confirm the system is performing correctly prior to CP injury experiments to ensure proper pressure readings. This is key as the ocular compliance of porcine eyes were found to be similar to the system with an air bubble present (Fig. 2B). It would be challenging in this case to separate the impact of the system and ocular tissue during puncture experiments. Conversely, when the system is properly setup, the system compliance was less than 5% of the ocular compliance value.

Next, the outflow rate when an enucleated porcine eye is connected, was measured by recording the pressure decay vs. time from 20 mmHg to 15 mmHg. Using a previously developed mathematical relationship^15^, outflow facility was determined to be approximately 0.71 µL/min/mmHg, differing slightly from previously reported outflow facility results of porcine tissue, which range from 0.24 to 0.61 µL/min/mmHg (Fig. 2C)^16–18^. Multiplying the outflow rate times normal IOP (15 mmHg), or the upper limit of IOP values reached during compliance testing (40 mmHg), an overall leak rate can be calculated (Fig. 2D). Comparing this to the 20 µL/s syringe pump infusion rate reveals that the outflow rate is insignificant by comparison, across the IOP operating range. Overall, these results provide simple metrics to confirm the system and connected ocular tissue are performing correctly prior to testing, increasing the CP injury model reliability and overall consistency.

### Impact of tissue preservation on ocular properties

**Figure 3:**
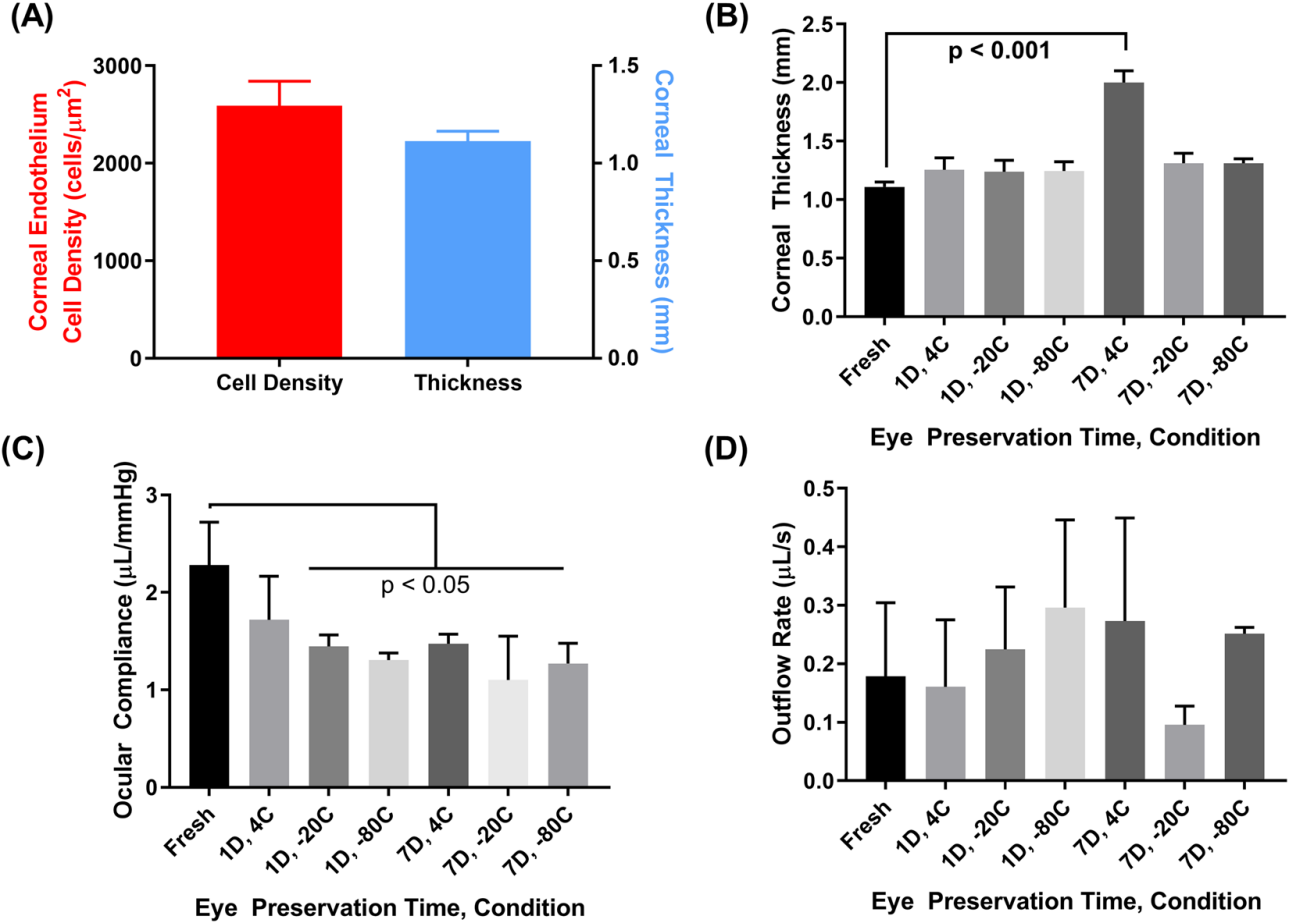
The effects of storage conditions on porcine tissue properties. (**A**) Corneal endothelium density of fresh ocular tissue (n = 19 biological replicates, left axis) as determined by specular microscopy and corneal apex thickness of fresh ocular tissue (n = 20 biological replicates, right axis) as determined by optical coherence tomography (OCT). (**B**) Comparison of corneal thickness for fresh tissue vs. tissue stored at 4 °C, −20 °C, −80 °C for 1 or 7 days. Statistical significance determined by one-way ANOVA, post-hoc Dunnett’s multiple comparison test. (**C**) Comparison of ocular compliance for fresh vs. preserved tissue after 20 µL volume injection. Statistical significance determined by one-way ANOVA, post-hoc Dunnett’s multiple comparison test. (**D**) Outflow rate at 15 mmHg for fresh vs. preserved tissue (n = 3 biological replicates for each). Statistical significance determined by Kruskal-Wallis test, post-hoc Dunn’s multiple comparisons test. Error bars denote standard deviation.

The next steps for standardizing the CP injury model were investigating whether porcine tissue obtained within 24 hours post-mortem have ocular properties similar to literature values and whether tissue properties could be preserved for up to 7 days post-mortem. We examined corneal endothelial density and central corneal thickness which were found to be approximately 2600 cells/mm^2^ and 1.1 mm, respectively (Fig. 3A). The variability between tested tissue was minimal and these results prove similar to reported porcine endothelial densities, ranging from 2500–4000 cells/mm^2^ and corneal thickness, ranging from 0.8 to 1.2 mm^19–23^. For assessing tissue storage, we assessed eyes 1 or 7 days after storage at 4 °C (submerged in HBSS), −20 °C (wrapped in HBSS soaked gauze), and −80 °C (wrapped in HBSS soaked gauze) (Table 1 for experimental group layout). An increase in corneal thickness occurred for all storage conditions, as compared to fresh tissue, by approximately 0.1 mm. There was a statistically significant difference in corneal thickness after 7 days storage at 4 °C (Fig. 3B). Ocular compliance was decreased in all preservation conditions, with all conditions being significantly reduced with the exception of 1 day, 4 °C (Fig. 3C). Lastly, outflow rate at 15 mmHg was similar across varying storage conditions with only 7 days storage at −20 °C trending lower than fresh tissue (Fig. 3D). Overall, these results highlight the importance of maintaining tissue freshness when performing corneal puncture injuries due to storage and preservation effects on key ocular properties.

### High-speed corneal puncture platform characterization

**Figure 4:**
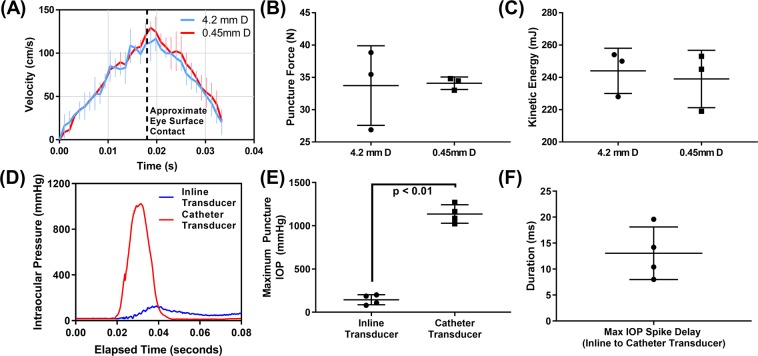
Characterization of Corneal Puncture Process. (**A**) Quantification of solenoid device instantaneous velocity vs. time immediately after firing as determined from high speed video footage. Velocity was quantified for 3 technical replicates with a 4.2 mm and 0.45 mm diameter object attached. Vertical dashed line represents the travel distance when the puncture object would make contact with the eye. Note the solenoid device contains a retraction spring which results in deceleration. Quantification of (**B**) force and (**C**) kinetic energy for 2 CP object sizes at the travel distance when contact with the eye would occur (n = 3 technical replicates). No significant differences between the object sizes were found. (**D**) Pressure vs. time traces for the catheter pressure transducer and in-line transducer connected by anterior needle cannulation following contact with the 2.4 mm diameter CP object at 0.02 seconds. (**E**) Quantification of the maximum IOP recorded during the CP injury process for the in-line transducer and catheter transducer (n = 4 biological replicates). (**F**) Quantification of the difference between the times (milliseconds) at which maximum IOP was measured by the in-line and catheter transducers (n = 4 biological replicates). Statistical differences between the two groups was determined by Student’s t-test, where p < 0.05 denotes statistical significance.

Prior to assessing CP injuries in ocular tissue, the high-speed solenoid device was characterized in terms of its dynamics after firing. Representative video footage of the solenoid device firing is shown in Supplementary Video 1. For quantifying puncture velocity, high speed video footage was captured perpendicularly to eliminate any three-dimensional artifacts that would skew quantification of video footage. We found that the velocity profile for the smallest CP object (0.45 mm diameter) and largest CP object (4.2 mm diameter) were similar, due to the majority of the projectile mass coming from the solenoid shaft and drill chuck instead of the puncture object (Fig. 4A). Velocity of the projectile reached approximately 120 cm/s prior to contacting the cornea. The device deaccelerates rapidly due to compression of a return spring on the solenoid device. The force and kinetic energy at the ocular surface as the CP was created were approximately 34 N and 240 mJ (Fig. 4B,C). These metrics were consistent for both the largest and smallest CP object size.

### Importance of pressure transducer location for CP injury model

Two pressure transducers are present in the benchtop model: a fluidic in-line pressure transducer connected by a needle placed in the anterior chamber (approximately 60 cm linear distance of tubing between eye and pressure transducer) and a catheter pressure transducer drawn into the vitreous cavity. When puncturing an eye with the benchtop model, the pressure vs. time plots across the approximate 40 ms after injury initiation, dramatically differ for the two transducers (Fig. 4D). With a 2.4 mm diameter CP object, the maximum pressure for the catheter transducer and in-line transducer were 1140 mmHg and 145 mmHg, respectively, producing an 8-fold difference in reading (Fig. 4E). Interestingly, the maximum pressure recorded by the in-line transducer was consistently delayed by approximately 10 ms from the catheter maximum, suggesting a pressure propagation delay on this time scale for the transducer located outside the eye (Fig. 4F). In addition, there was increased noise in the raw pressure trace data for the in-line transducer relative to the catheter transducer, likely due to how the CP puncture process causes significant motion to the anterior chamber needle (Supplementary Video 2,3). As a result, while the in-line transducers reading are essential when setting up the benchtop platform, the catheter transducer produces more reliable IOP readings during CP injury creation.

### Effect of CP object size on CP injury characterization

**Figure 5:**
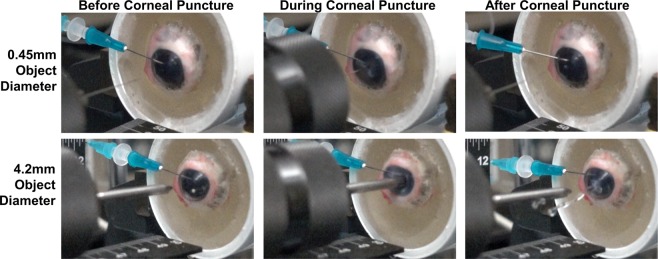
Corneal Puncture Process. Representative images immediately before (**Column 1**), during (**Column 2**), and after (**Column 3**) CP object contact with the eye after firing from the high-speed solenoid device for a 0.45 mm (**Row 1**) and 4.2 mm (**Row 2**) CP object diameter.

**Figure 6:**
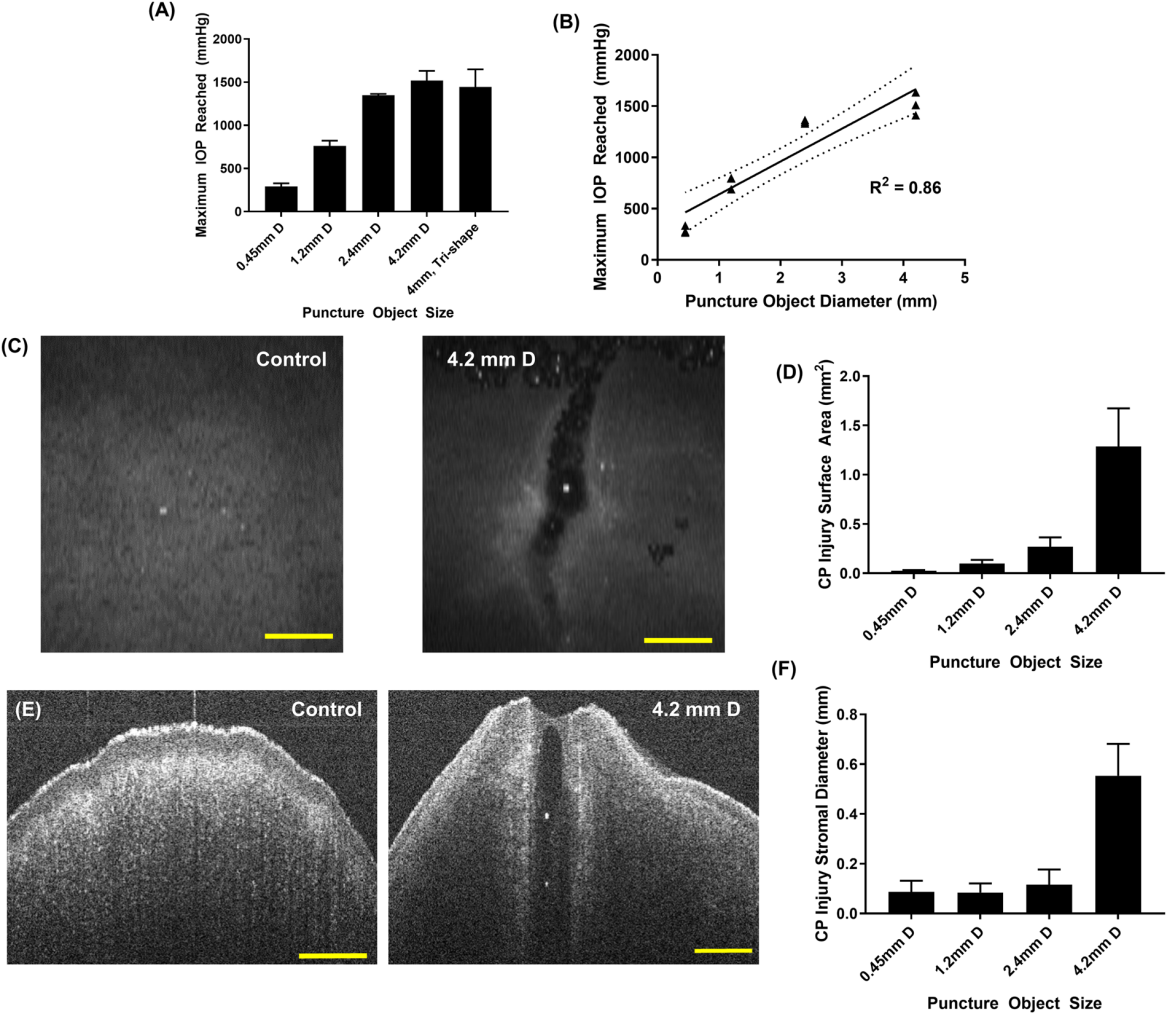
Characterization of various CP injury sizes created using benchtop model. (**A**) Maximum IOP recorded by catheter pressure transducer for different CP object sizes and shapes (n = 3 biological replicates for each). (**B**) Maximum IOP vs. object diameter correlation plot and resulting linear regression. Dotted lines represents 95% confidence intervals for the regression analysis, and the coefficient of determination (R^2^) is shown. Triplicate data points are shown for each object diameter tested. (**C**) OCT volume projection image of the anterior corneal surface for a control eye (left) or after CP injury with 4.2 mm diameter object (right). (**D**) Surface area (mm^2^) of the CP injury from volume projections for different CP injury sizes. (n = 3 biological replicates). (**E**) Sagittal or cross-sectional view for a control eye (left) or after CP injury with 4.2 mm object diameter (right). (**F**) Approximate diameter (mm) of the CP injury through the corneal stroma for different CP injury sizes (n = 3 biological replicates). Scale bars denote 1 mm. Error bars denote standard deviation.

Lastly, we characterized how the injury model can be used to create high-speed CP injuries of a variety of shapes and sizes. The smallest CP object, 0.45 mm diameter, and largest CP object tested, 4.2 mm diameter, both successfully punctured porcine tissue consistently, however the force required and, thus, pressure generated, were noticeably different from captured high-speed video footage (Fig. 5, Supplementary Video 2 [0.45 mm diameter], Supplementary Video 3 [4.2 mm diameter]). The catheter pressure readings reflect this observation, with the smallest object generating approximately 300 mmHg IOP while the largest CP object resulted in IOP exceeding 1500 mmHg (Fig. 6A). Comparing the maximum pressure and object diameter resulted in a significant linear correlation, with a coefficient of determination (R^2^) of 0.86 (Fig. 6B). Interestingly, two objects of similar shaft diameter (4.2 mm vs. 4 mm diameter) with different puncture head geometry (conical vs. star-shaped, respectively) resulted in similar IOP maximums (Fig. 6A).

CP injuries were imaged by OCT, and volume intensity projections (Fig. 6C) were used to quantify the corneal epithelium surface area of the CP injury site. Sagittal views through the cornea (Fig. 6E) were used to quantify an approximate puncture diameter through the corneal stroma. The overall surface area and diameter differed across CP object sizes and trended directly with CP object diameter. This was more apparent at the surface area (Fig. 6D) than from diameter estimates, likely due to a larger, more noticeable area being easier to quantify (Fig. 6F). Overall, we were able to characterize the CP injury model and show that a variety of injury sizes can be created.

## Discussion

In recent combat operations corneal puncture injuries resulted in poor visual outcomes for injured warfighters, often resulting in blindness. This is predominately due to inadequate battlefield stopgap measures to close open ocular wounds and restore IOP. This is a critical medical shortcoming and development of therapeutics is inhibited by there being no military relevant injury test platforms available. Unlike civilian CP injuries, military CP injuries occur most notably from improvised explosive devices (IEDs), which create irregularly shaped injuries from high-speed shrapnel that have an average width of 3.5–5 mm^3,4,7^. It is key to mimic this unique situation in order to properly develop CP therapeutics. Results from this study take first steps towards solving this capability gap, by developing a benchtop open globe injury model for use with excised tissue.

Our developed benchtop model utilizes a high-speed solenoid device fitted with a drill chuck to create injuries at military relevant velocities, shapes, and sizes. The device at the point of injury travels at approximately 120 cm/s, requiring only a few milliseconds to fully puncture the eye. Tissue mechanical properties differ with increasing velocities, therefore a slow-speed scalpel based incision, often used in other studies to create CP injuries, are not an effective comparison to the high speed injuries on the battlefield^24–27^. Furthermore, our model accommodates objects up to 10 mm in diameter, with the largest CP object tested to date being 4.2 mm in diameter. The pressure generated from objects of this size were different than small injuries, with IOP reaching as high as 1500 mmHg. Lastly, since we were able to utilize large objects that were less sharpened than conventional scalpels, the geometry of the CP injury created was irregular, in line with military relevant injuries. The injury site was often elongated in one axial direction, indicating CP injuries were propagating in a ripping motion.

As we developed the benchtop CP model, each aspect of the model was evaluated and standardized to provide baseline performance metrics where necessary. We standardized the puncture travel distance into the eye at 16 mm as that distance allowed for consistent CP injury while damage to intraocular structures was not qualitatively detected. This distance is greater than the depth of the anterior chamber, but due to compression of the gelatin and ocular tissue during the puncture process, this distance was needed to consistently penetrate the cornea. Damage to intraocular structures was only determined by whether the iris or lens were punctured, as intraocular damage is less of a concern with benchtop testing where explant tissue is only assessed for minutes to hours after CP injury. Further assessment over days to weeks will be needed for transitioning this model toward animal studies, as internal damage may present confounding factors in the model. The effect of tissue storage conditions on ocular properties was also evaluated as it is often challenging to procure and test tissue within a 24 hour time frame. Unfortunately, the mechanics of the tissue were altered in all scenarios tested with the cornea thickening and ocular compliance decreasing. Increased corneal edema is to be expected as the corneal endothelium breaks down^28–30^, and tissue stiffening with preservation has been observed in other tissue types^31–33^. One limitation for the benchtop model is that testing is performed at room temperature due to the low melting point of gelatin used to hold the eye; measured mechanics may differ when testing at physiological temperatures. Lastly, we developed baseline measurements for outflow facility, ocular compliance and overall system compliance that should be evaluated prior to tissue testing. This ensures that tissue is relatively comparable between experiments and the system is functioning correctly, both of which are essential for comparing results across experiments. However, outflow facility was found to differ from previously published results looking at porcine outflow facility. This was likely due to previous reports utilizing systems exclusively measuring outflow under constant pressure, variable flow rate^16–18^. Alternatively, the benchtop CP model relied on variable flow and constant pressure, along with 2 needles cannulating the eye, including a 16 gauge needle placed in the vitreous chamber, likely altering the outflow rate. With all the steps taken toward standardizing the CP injury model, the average coefficient of variation of biological replication for maximum IOP generated from different CP injury sizes was only 0.087, exemplifying the consistency of this model.

While characterizing the model, one interesting finding was the magnitude of IOP reached as the cornea was punctured. We observed IOP over 1500 mmHg for our largest injury size and still almost 300 mmHg for a small, 0.45 mm diameter injury. The extent of intraocular damage that results from IOPs at this magnitude have not been characterized in this model. It is known that chronic elevated IOP for acute injuries to the eye, may result in different forms of glaucoma^34–36^. However, the IOP spike in these scenarios only lasts 10–20 milliseconds, after which pressure returns to atmospheric, due to the nature of the open-globe injury. This may be similar to rapid IOP spikes from blinking (10 to 20 mmHg increase) or eye rubbing (100 to 200 mmHg increase) that rarely result in ocular damage^37^. However, given that the IOP spike is much greater, there is potential for disruption of aqueous humor inflow or trabecular meshwork/Schlemm’s canal outflow pathways that could result in long-term ramifications of this CP injury^38–40^. Further work is needed tracking long-term damage following open-globe injuries, especially given that the average time between treatment and injury for CP injuries with IOFB present was 21 days in recent combat operations^4^.

While this model represents a positive first step towards developing therapeutics for military-relevant corneal injures, there are some limitations with the current approach. Using enucleated tissue CP injuries can only be tracked for a few hours after injury creation. For assessing multi-day or multi-week time courses an alternative approach will be needed where anterior tissue can be maintained long term post-enucleation, such as in organ culture^41–45^, or by transitioning to an animal model. Another limitation is the eye holder housing the eye in gelatin is uniformly shaped and thus does not exactly mimic the complex geometry of the ocular orbit. Distributions of loads and generated pressure may differ when translating to an *in vivo* setup. Other limitations include the force and speed of our current device. While the force and speed generated by our high-speed solenoid device was more military relevant than scalpel-based injuries, it was still orders of magnitude slower than projectile velocities seen with IED devices^46,47^, which are the most common cause of CP military injuries^48^. There are approaches that can be taken to further approach IED speeds, but may prove challenging to incorporate into a user friendly benchtop model format.

In conclusion, the developed benchtop CP injury model is capable of producing military relevant injuries that can be used for therapeutic testing or further injury characterization. This model provides a simplistic approach to conduct preliminary testing of therapeutics and puncture wound sealants in excised ocular tissue and provides an alternative approach for reducing animal studies. Our results showed that our CP injury platform is highly consistent, once baseline parameters were established for the system and ocular properties were evaluated prior to using the model to confirm comparable performance. With this key step established, we characterized CP injury sizes up to 4.2 mm in diameter, similar to the average injury size in military cases. Interestingly, the pressure generated in such injuries surpassed 1500 mmHg, approximately 100x physiological pressure, which may result in damage to other ocular structures. Future work will look at evaluating potential corneal adhesives for sealing or treating CP injuries, and transitioning this benchtop model into an animal or *ex vivo* platform for long term tissue testing after injury.

## Methods and Materials

### Tissue sourcing

Porcine eyes (Animal Technologies, Tyler, TX) were enucleated and kept in phosphate buffered saline (PBS) on ice. Connective tissue was removed from eyes leaving the globe intact prior to use. For testing tissue storage conditions, eyes were divided into 7 groups (Table 1) based on their storage temperature or duration. Briefly, eyes were used within 24 hours post-mortem (fresh), stored at 4 °C in Hanks’ balanced salt solution (HBSS), stored at −20 °C wrapped in HBSS-soaked gauze, or stored at −80 °C wrapped in HBSS-soaked gauze for 1 or 7 days. Eyes were thawed for 3 hours at room temperature prior to testing.

**Table 1:** Experimental groups for evaluation of storage conditions for porcine ocular tissue.

Day 0, no storage	24 hours (Day 1) storage	168 hours (Day 7) storage
Used within 24 hours post mortem. Kept on ice. (Group 1)	4 °C, stored in HBSS (2)	4 °C, stored in HBSS (5)
	−20 °C, wrapped in HBSS-soaked gauze (3)	−20 °C, wrapped in HBSS-soaked gauze (6)
	−80 °C, wrapped in HBSS-soaked gauze (4)	−80 °C, wrapped in HBSS-soaked gauze (7)

### Benchtop corneal puncture injury model

The benchtop CP injury model uses a high speed solenoid device (Fig. 1A, MurCal, and Palmdale, CA) capable of generating up to 60 N of force. The solenoid was fitted with a drill chuck (McMaster-Carr, Elmhurst, IL) which was capable of holding CP objects up to 10 mm in width. Porcine eyes were placed in a device holder (Fig. 1B) which housed a 16 gauge needle for cannulating the posterior eye (Fig. 1C) and secured the eye using 10% w/w gelatin, which has previously been shown to mimic extraorbital tissue properties (Sigma-Aldrich, St. Louis, MO)^49–51^. To secure the eyes, gelatin solution was placed in each device holder and a custom 3D-printed curved mold (Ultimaker 3, Utrecht, Netherlands) was positioned to create an impression that will be used to place the eye. After the gelatin solidified for 30 minutes at 4 °C, the curved mold is removed and the central posterior eye was mounted on the 16-gauge needle (6 mm penetration depth through the posterior sclera), and additional gelatin solution was added up to the limbal region, followed by further gelation for 15 minutes at 4 °C.

The eye holder was mounted horizontally with a bench-vise (McMaster-Carr, Elmhurst, IL), and cannulated by 23 gauge needle connected to a syringe pump (Fig. 1D, KD Scientific, Holliston, MA), in-line pressure transducer (Fig. 1E, Model: MLT844, AD Instruments, Sydney, Australia), and hydrostatic reservoir (Fig. 1F) so that volume can be adjusted, pressure can be monitored and intraocular pressure (IOP) can be set, respectively. A secondary pressure transducer was connected using the 16-gauge needle inserted into the vitreous chamber and a Mikro-Tip pressure catheter (Model: SPR-524, Millar, Houston, TX) was drawn into the posterior eye 16 mm (10 mm beyond the 16-gauge needle).

Prior to use, both pressure transducers were calibrated using hydrostatic reservoirs to determine linearity from 0 to 75 mmHg, the typical operating range. The catheter pressure transducer’s linearity exceeded 1000 mmHg, as determined by recording the force (Univert Mechanical Testing System, CellScale, Waterloo, ON, Canada) exerted on a known surface area and recording the pressure response in a closed system (data not shown).

### Benchtop CP model stability assessment

Prior to each usage, the CP model was characterized to determine the system was closed and does not contain compressible air bubbles in the tubing or valves that will drastically alter pressure readouts (LabChart, AD Instruments, Sydney, Australia). After zeroing both pressure transducers by placing the hydrostatic reservoir at 0 mmHg, 20 µL of saline was infused at 20 µL/s into the system while it was closed to the hydrostatic reservoir or the eye (syringe pump, system tubing, and in-line pressure transducer only), and the pressure response was recorded (100 readings/second). The result was compared to baselines for the system to determine if the pressure response to the volume injection was comparable. If not comparable, the system was inspected to remove any air bubbles or tighten any fluidic components. Testing was repeated until trends were consistent with baseline readings.

### Benchtop CP model system characterization

To characterize the fluidic system required for recording pressure, system compliance was measured and compared to ocular compliance. Compliance is a mechanical property defined as the change in volume for a given change in pressure ($\frac{\Delta V}{\Delta P}[=]\frac{\mu L}{mmHg}$)^15^. System compliance was measured in a closed-system with the in-line pressure transducer and system fluidics isolated, and 20 µL being injected via syringe pump at 20 µL/s. To account for fluidic resistance in the system, injections were performed as described above, while the system remained open. Ocular compliance was measured in an identical manner, except with the system attached to a porcine eye. Volume injections of 20 µL at 20 µL/s were introduced and the in-line and catheter pressure transducers results were captured. Volume injections were repeated three times, with the initial pressure starting at approximately 15 mmHg each time.

Next, outflow rate was determined based upon previously described methods^15^. Pressure was set at 20 mmHg by hydrostatic reservoir, the system was isolated from the reservoir, and pressure versus (vs.) time data were captured until pressure readings reached 15 mmHg. This pressure vs. time relationship was fitted using Excel Solver to a previously described exponential relationship:

1 $$P(t)={K}_{1}+{K}_{2}{e}^{-t/\tau }$$

Where *P*(*t*) is the recorded pressure in mmHg, t is the corresponding time in seconds, and ${K}_{1},{K}_{2},\tau$ are fitting parameters. Outflow facility, or the change in outflow rate per corresponding IOP, can be calculated as:

2 $$Outflow\,facility\,[=]\frac{\frac{\mu L}{min}}{mmHg}=\frac{Complianc{e}_{eye}+Complianc{e}_{system}}{\tau }$$

Outflow rate for a specific IOP is the product of IOP and outflow facility.

### Effect of tissue preservation on ocular mechanics

We next looked at tissue sourcing and the effects of long-term storage on the CP injury model. Fresh porcine tissue was characterized for corneal endothelial density, as measured by specular microscopy (NIDEK, Tokyo, Japan), and corneal thickness, as measured by optical coherence tomography (OCT, Bioptigen, Leica, Wetzlar, Germany). Additionally, outflow rate and ocular compliance were evaluated for comparison to previously reported literature to confirm similar readings. It should be noted that the fresh tissues used in these studies were obtained from the abbatoir within 24 hours post mortem. This timeframe introduced a possibility of some tissue swelling to occur. However, the measured thicknesses of the fresh corneas used in this study were in agreement with other published values of *in vivo* and freshly explanted porcine corneas. It is unlikely that swelling of the tissues is a concern in the studies presented here and that the eyes are a good representation of *in vivo* physiologic conditions. Preserved eyes (as described in Table 1) were correspondingly evaluated for comparison to fresh tissue in order to determine if preservation impacted any relevant ocular property. Due to corneal thickness changes and specular microscopy’s limited penetration depth, corneal endothelial density was not assessed in preserved tissue.

### Solenoid CP mechanical characterization

The solenoid device utilized for creating CP injuries was characterized by determining the optimal penetration depth into the eye required to consistently create a CP injury without puncturing the lens or iris. Using a 2.4 mm diameter CP object, a 13 mm to 18 mm penetration depth into the eye was qualitatively assessed based on its ability to create a CP injury without puncturing the lens or iris, which were assessed after dissection (data not shown). The optimal penetration depth, 16 mm, was repeated in three eyes to confirm consistent CP injury without puncturing the lens or iris.

Next, the firing of the solenoid device was recorded by 2 high-speed video cameras. For qualitative assessment of the puncture process, a camera (RX100 VI at 960 fps, Sony Corporation Tokyo Japan) was positioned behind the puncture device to observe CP injury across the corneal surface. For quantifying the velocity of the puncture process, high speed footage was captured (FASTCAM Mini at 6400 fps, Photron Cameras, Tokyo, Japan) perpendicular to the puncture device to remove any 3D artifacts. This was done with the smallest CP object, 0.45 mm diameter, or largest CP object, 4.2 mm diameter, attached. Using Tracker software v5.1.1 (Open Source Physics, Davidson, NC), the velocity and acceleration of the CP object were quantified during forward travel. Force and kinetic energy (KE) at the point of contact with the cornea were calculated as follows:

3 $$Force=m\ast a$$

4 $$KE=\frac{1}{2}m{v}^{2}$$

Where *m* is the mass of CP object, drill chuck, and solenoid inner shaft, *a* is acceleration, and *v* is velocity.

### CP injury process characterization

CP injuries were created in fresh porcine tissue using a 0.45 mm, 1.2 mm, 2.4 mm, or 4.2 mm diameter objects. These diameters are the shaft diameter as each object was conical shaped and came to a sharpened point (different size commercially available nails, except the 0.45 mm object sewing needle). In addition, a 4.0 mm diameter shaped object with a complex triangular puncture head was used to demonstrate the ability of the model to utilize irregular object shapes. Prior to injury, ocular compliance and outflow facility were measured to confirm tissue was consistent with determined baselines. IOP was then set at 15 mmHg. The puncture object was brought in direct contact with the corneal apex and withdrawn 12 mm (controlled via Labview, National Instruments, Austin, TX), leaving 16 mm travel distance into the eye (28 mm total solenoid travel distance). While recording pressure (10 kHz) and capturing the process with high-speed video, the solenoid device was fired. Next, outflow rate was measured to verify an instantaneous loss of pressure, confirming open-globe injury. Eyes were fixed with 10% buffered formalin, and the injury geometry was assessed by OCT.

### Statistics

For all porcine tissue experiments, at least three eyes were used and exact biological replicates are indicated throughout. For system or ocular compliance, three technical replicates were measured and averaged. All statistical analysis was performed using GraphPad Prism 8.1.2 (La Jolla, CA, USA). For fitting experimental pressure vs. time data to a mathematical approximation (estimated using Excel Solver), a Pearson’s correlation between experimental and modeled pressure was used to confirm parameter fit, where p < 0.05 indicated a significant correlation. Prior to parametric statistical analysis, the Brown-Forsythe test was used for assessing equal variance and Shapiro-Wilk test was used for testing if the sample came from a normally distributed population. When comparing two groups, Student’s t-tests were used as indicated throughout. For comparing multiple groups to a single control, one-way ANOVA analysis was performed with Dunnett’s post-hoc test. Alternatively, when parametric testing was not valid, Kruskal-Wallis test with Dunn’s multiple comparison post hoc test was used. Throughout, p < 0.05 indicated significant differences.

### Disclaimer

The views expressed in this article are those of the author(s) and do not reflect the official policy or position of the U.S. Army Medical Department, Department of the Army, Department of Defense, or the U.S. Government.

## Supplementary information

Supplementary Video 1.

Supplementary Video 2.

Supplementary Video 3.

## Data Availability

The datasets generated during and/or analyzed during the current study are available from the corresponding author upon reasonable request.
